# Larvicidal Effectiveness of* Azolla pinnata* against* Aedes aegypti* (Diptera: Culicidae) with Its Effects on Larval Morphology and Visualization of Behavioural Response

**DOI:** 10.1155/2018/1383186

**Published:** 2018-06-27

**Authors:** Nor Shaida Husna Zulkrnin, Nurul Nadiah Rozhan, Nur Amanina Zulkfili, Nik Raihan Nik Yusoff, Mohd Sukhairi Mat Rasat, Nor Hakimin Abdullah, Muhammad Iqbal Ahmad, Rajiv Ravi, Intan H. Ishak, Mohamad Faiz Mohd Amin

**Affiliations:** ^1^Faculty of Earth Science, Universiti Malaysia Kelantan, Jeli Campus, Jeli, Kelantan, Malaysia; ^2^Faculty of Bioengineering and Technology, Universiti Malaysia Kelantan, Jeli Campus, Jeli, Kelantan, Malaysia; ^3^School of Biological Sciences, Universiti Sains Malaysia, Minden, Penang, Malaysia; ^4^Vector Control Research Unit, School of Biological Sciences, Universiti Sains Malaysia, Minden, Penang, Malaysia

## Abstract

Dengue is vector-borne diseases with 390 million infections per year extending over 120 countries of the world.* Aedes aegypti* (L.) (Diptera: Culicidae) is a primary vector for dengue viral infections for humans. Current focus on application of natural product against mosquito vectors has been the main priority for research due to its eco-safety. The extensive use of chemical insecticides has led to severe health problems, environmental pollution, toxic hazards to human and nontarget species, and development of insecticide resistance on mosquitoes.* Azolla pinnata* is an aquatic fern and predominantly used as feed in poultry industry and as fertilizer in agricultural field for enhancing the fertility of rice paddy soil. The present study was conducted to explore the larvicidal efficacy of* A. pinnata* using fresh and powdered form against late third-stage larvae (6 days, 5 mm in larvae body length) of* Ae. aegypti* (L.) (Diptera: Culicidae). The larvicidal bioassays were performed using World Health Organization standard larval susceptibility test method for different concentration for powdered and fresh* A. pinnata.* Powdered* A. pinnata* concentration used during larvicidal bioassay ranges from 500ppm to 2000ppm; meanwhile, fresh* A. pinnata* ranges from 500ppm to 9,000,000 ppm. The highest mortality was at 1853 ppm for powdered* A. pinnata* compared with fresh* A. pinnata* at 2,521,535 ppm, while the LC_50_ for both powdered and fresh* A. pinnata* recorded at 1262 ppm and 1853 ppm, respectively. Finally, the analysis of variance (ANOVA) showed significant difference on* Ae. aegypti* larval mortality (F=30.439, df=1, p≤0.001) and concentration (F=20.002, df=1, p≤0.001) compared to powdered and fresh* A. pinnata* at 24-hour bioassay test. In conclusion, the powdered* A. pinnata *serves as a good larvicidal agent against* Ae. aegypti* (L.) (Diptera: Culicidae) and this study provided information on the lethal concentration that may have potential for a more eco-friendly* Aedes* mosquito control program.

## 1. Introduction

The intense increase on arthropod-borne viruses (arboviruses) diseases such as dengue, Zika, and Chikugunya is mainly contributed by primary vector* Ae. aegypti*. Recently in Malaysia, from January till July 2017 there were 55,744 dengue cases with 131 deaths [1]. Previously, Ishak [2] reported that 50 million vector-borne disease cases worldwide were due to dengue and Malaysia has reported 46,171 cases in 2010 with 134 deaths. In addition to that, the planning of infrastructure development and management has also contributed in arthropod-borne viruses (arboviruses) diseases. World Health Organization [3] and Mohd Amin [4, 5] have stated that unplanned urbanization with the defect in water supply and solid waste management's have mainly contributed in arboviral diseases spread by the mosquitoes. Currently, the control measures of dengue vector in Malaysia are adulticiding with permethrin, deltamethrin, and malathion and larviciding using temephos and* Bacillus thuringiensis israelensis* (*Bti*) [2]. Additionally, Malaysian Ministry of Health (MoH) operators, private companies, and household communities are using insecticides to control dengue vectors. However the intense exposures from all this usage have contributed to insecticide resistance in Malaysian* Aedes* populations. In Malaysia, evidence of resistance towards permethrin and temephos has been recorded from both* Ae. aegypti* and* Ae. albopictus* in Kuala Lumpur and Penang regions [6, 7]. Ishak [2] has reported that insecticide resistance was caused by two main factors due to increase in rate of insecticide metabolism and alterations in its target sites. Additionally, alterations in target sites are caused by mutations from target genes such as knockdown (*kdr*) resistance, acetylcholinesterase (*Ace-1*) gene, and GABA receptors [2].

Hence due to all these problems in controlling dengue vector, alternative method is required. In such situation, alternative usage of biological control can provide a more suitable and sustainable solution against* Ae. aegypti.* Following this safer and greener alternative conception,* Azolla pinnata* plant has the potential as bioinsecticide may solve the problems of resistance and chemical pollution. In addition to that, this conception was also supported by Ghosh [8], who mentioned that bioinsecticide from botanical origin is simple and sustainable method compared to conventional insecticides. Unlike conventional insecticides, advantages of plant-derived insecticides which is composed by botanical blends of chemical compounds will act concertedly on both physiological and behavioural processes [8]. Hence, there would be only very minute possibilities of vectors in developing resistance to such substances.

One of the first field studies from genus of* Azolla* plant for its effects on mosquito breeding was by Bao-Lin, L [9], from Hunan, China, on* Azolla filiculoides* which has reduced the larval density of* Culex tritaeniorhynchus* by an average of 69% for 3 months at 75% coverage of water surface at 800 hectare of paddy land. Moreover, reduction of* Anopheles sinensis* larval densities was not clear in his observations, but the pupal densities were reduced for both larval species [9]. On the other hand, another field study report with* A. pinnata *as mosquito breeding control was by Pandey [10] from Gujerat, India. It was mentioned that* A. pinnata* has an effect on the oviposition of* Anopheles culicifacies* and* An. subpictus* mosquitoes in rice fields [10].

Similarly, Pandey [10] have reported a lab based study with* A. pinnata* which affects the oviposition of* Culex quinquefasciatus* and* Cx. culicifacies *mosquitoes. Lab containers covered with 50% of* A. pinnata* result in a suppression of egg laying properties with its significant behavioural changes on* Cx. quinquefasciatus *and* Cx. culicifacies* mosquitoes [10]. Additionally, in paddy fields of Tanzania, Africa, Mwingira, V. S [11] found* Anabaena azollae* have reduced the larvae productivity and larvae densities of* An. gambiae, An. funestus*, and* Cx. quinquefasciatus.* Mwingira, V. S [11] findings suggest that the mosquito productivity is low when the Azolla coverage is high (>80%) in paddy fields.

Despite the fact of many studies on* Azolla* plant with mosquitoes, none of them have mentioned the use of fresh and powdered form of the plant and its direct applications on* Ae. aegypti*. However, understanding these future potentials, based on the papers reviewed, it seems that* A. pinnata* has bioinsecticides potentials with its alterations against behavioural changes in mosquito vectors. In addition to that, none of the papers have investigated the larvicidal, morphological, and behavioural efficacies on* Ae. aegypti*.

Hence, this study will be focused on* A. pinnata* fresh and powdered form as a biological control agent against* Ae. aegypti* larvae. To date, no other studies have been conducted with* A. pinnata* plant for its larvicidal efficacies and morphological and behavioural responses on* Ae. aegypti* larvae.

## 2. Material and Methods

### 2.1. Research Area and Design

This research follows World Health Organization, WHO [3] guidelines for* mosquito *larvicidal bioassay. A total of 50 kg fresh* A. pinnata* was sampled from Kuala Krai, Kelantan (5°31′N 102°12′E), and its species was identified based on leaves phyllotaxis morphological pattern [12].

### 2.2. Sample Preparation

#### 2.2.1. Powdered Samples

**Figure 1:**
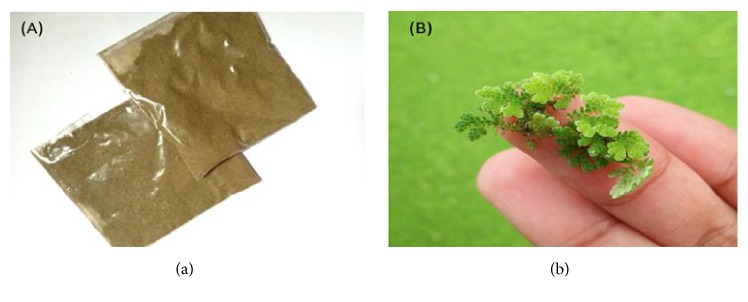
*Azolla pinnata* powder (a) and fresh* Azolla pinnata* (b).

Total of 30 kg,* A. pinnata* fresh sample was prepared using sun-dried technique for 2 days. Then the dried samples were powdered electrically with grinding machine, Faber FBG-460K and sieved as fine powder. Next, the* A. pinnata* powder was been stored in zipper bag and kept at room temperature (Figure 1).

#### 2.2.2. Fresh Samples

Fresh* A. pinnata* plant larvicidal tests were conducted with 20 kg of fresh plant. The fresh samples were then washed with chlorine free water to remove any impurities from other sources before using it for larvicidal bioassay (Figure 1).

### 2.3. Aedes Larvae Rearing

Susceptible lab strain eggs of* Ae. aegypti* were obtained from Vector Control Research Unit (VCRU) at University Sains Malaysia (USM), Penang, Malaysia. Then, the eggs were hatched in seasoned water for 24 hours. The hatching process was triggered with 0.2 g of larval food (food ratio; 2:1:1 of cat biscuit, beef liver, yeast and milk powder). The eggs were maintained at 25°C to 30°C (room temperature), a pH of 6.95 to 7.03, and relative humidity of 80 ± 10% and dissolved oxygen from 5.5 to 6.1 mg/L in the laboratory. After 6 days, the larvae turned into instars third stages.

### 2.4. Larvicidal Bioassay

Larvicidal bioassays were performed in accordance with the standard World Health Organization [1, 3] larval susceptibility test methods (distilled water and plant solution). The bioassay tests were done with four replicates of* Ae. aegypti* late third-stage larvae (6 days, 5 mm in larvae body length). Plastic containers (each contains; 500 mL of distilled water,* A. Pinnata* powder and 20 mosquito larvae) were used in this test [1]. After 24 hours, the mortalities of* Ae. aegypti* larvae were determined [1]. Larvae with total absence of movement, even after touch, were considered as dead.

### 2.5. Morphological View and Visualization

*Ae. aegypti* late third-stage larvae was observed under the optical microscope (Leica USA), magnification 40-400x. Visualization was conducted by Samsung ES80 camera.

### 2.6. Data Analysis

The percentages of mortality were subjected to log-probit analysis for computing LC_50_ and LC_90_ with 95% confidence limit using the SPSS 20.0 (Statistical Package of Social Sciences) software. Analysis of variance (ANOVA) was performed using the concentration and mortality between powdered and fresh in 24-hour bioassay test. Homogeneity of variance was tested using (Shapiro-Wilk) prior to analysis according to guidelines provided by Andy [13]. 24 hours of experimental time was served as independent variable and concentration and mortality were treated as dependent factors.

## 3. Results

**Figure 2:**
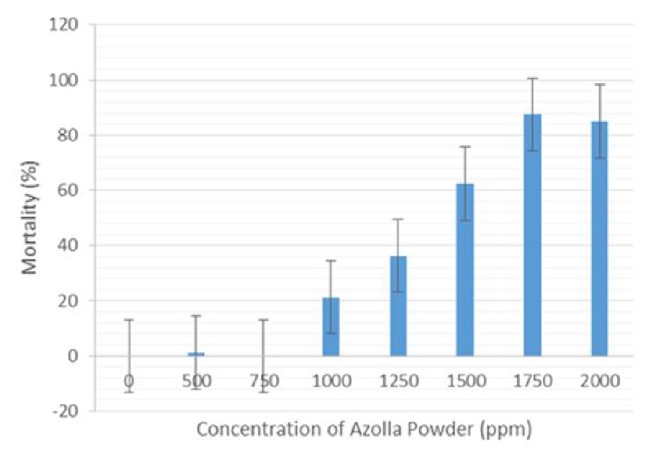
Percentage mean mortality of late third-stage larvae of* Ae. aegypti* after 24 hours in response to powdered* A. pinnata*.

**Figure 3:**
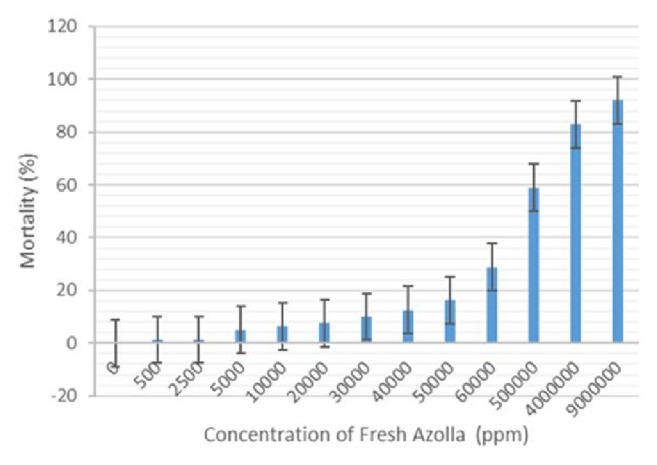
Percentage mean mortality of late third-stage larvae of* Ae. aegypti* after 24 hours in response to fresh* A. pinnata*.

The bioassay testing was conducted on critical range of 5% to 95% mortality for powdered* A. pinnata* in 500 ppm, 750 ppm, 1000 ppm, 1250 ppm, 1500 ppm, 1750 ppm, and 2000 ppm and fresh* A. pinnata* consisting of 500 ppm, 2500 ppm, 5000 ppm, 10000 ppm, 20000 ppm, 30000 ppm, 40000 ppm, 50000 ppm, 60000 ppm, 500,000 ppm, 4,000,000 ppm, and 9,000,000 ppm concentrations (Figures 2 and 3). The results showed a significant increase in mortality percentage with the increase of concentration in both cases. The highest mortality was demonstrated at 1853 ppm for powdered* A. pinnata* compared with fresh* A. pinnata* at 2,521,535 ppm (Table 1). Meanwhile the LC_50_ of powdered and fresh* A. pinnata* was recorded at 1262 ppm and 1853 ppm, respectively (Table 1).  Table 2 shows the annova results of* Ae. aegypti* larval mortality between powdered and fresh* A. pinnata* exposure for 24 hours.

**Figure 4:**
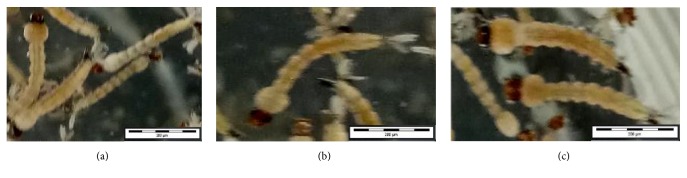
Morphological deformities and melanisation effects of* Ae. aegypti* late third-stage larvae after 24 hours in response to powdered and fresh* Azolla pinnata*.

**Figure 5:**
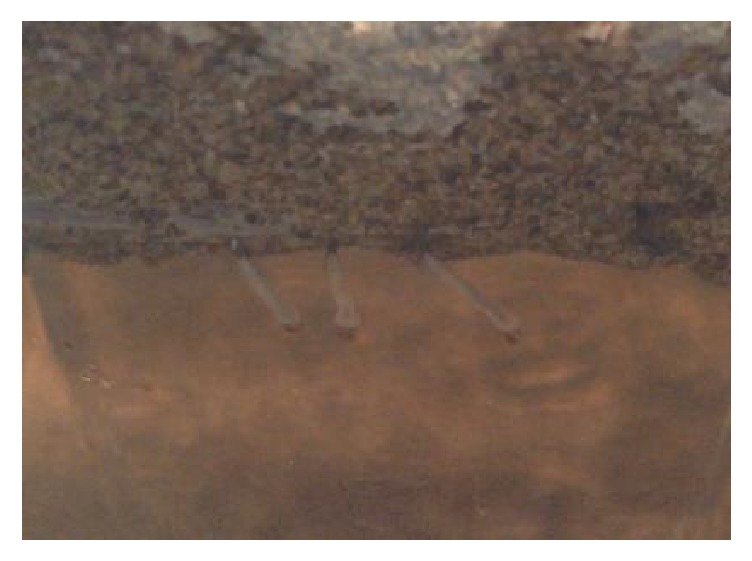
Visualization video captured on behavioural response effects of* Ae. aegypti* late third-stage larvae after 24-hour bioassay test in powdered and fresh* Azolla pinnata*.

Figures 4 and 5 show morphological deformities, melanisation, and behavioural effects of* Ae. aegypti* larval during the bioassay test; meanwhile the attached supplementary video (available here) file could intelligible to their behavioural responses. Hence, it can be assertive that fresh and powdered* A. pinnata *acts as a biological control against* Ae. aegypti*.

## 4. Discussion

This study has demonstrated the biocontrol efficacies of fresh and powdered* A. pinnata *against* Ae. aegypti* late third-stage larvae. Additionally, the* Ae. aegypti* late third-stage larvae show some morphological deformities and abnormal behavioural responses during these bioassays. Interestingly, the physical morphological observation of the larvae during 24 hours of exposure showed a brownish colour abdominal segment which changes into whitish colour (Figure 4). As shown in Figure 5 and visualization videos, it could be further discussed as toxic accumulation in body effects upon* A. pinnata *applications. These are due to the adaptation on new environment, whereby the larva possesses passive movement and swims at the bottom, showing sluggish and wiggling behavioural responses. In addition to that, the larvae were flexing to clean their siphon with mouthparts which led to high possibilities of toxic accumulation in their body.

Lakshmi Naidu [14] reported that plant produces a broad range of bioactive chemical compounds consisting secondary metabolites such as flavonoids, tannins, terpenoids, and alkaloids which would significantly produce biological activities and chemical defences against insects. Hence,* A. pinnata* might have contributed in body effects of larvae, due to its natural bioactive chemical compounds and secondary metabolites. Results from this current study indicate the potential application from* A. pinnata* plant as mosquito larvicidal agent. This is, because even-through low concentrations, we could observe larvae mortalities. Mogi [15] and Baolin [9] showed the effects of* Azolla* by covering the total water surface which affects the mosquitoes breeding, barrier on oviposition, and emergence of pupae, further reducing the larvae densities in rice fields.

Comparison of efficacies between powdered and fresh* A. pinnata* plant from this present study has shown that powdered application is the best in lab scale experiment. Similarly, Rao [16] have applied the neem cake powder which resulted in drastic reduction of late-instar larvae and pupae of Culicine mosquitoes from paddy fields compared to fresh plant application. Thus, this current study would be an important framework in the future investigation of bioinsecticidal from* A. pinnata* plant used for larvicidal test.

In conclusion, discovering an environmentally friendly insecticide to control mosquito vectors is considered to be a vital role which reduces the negative impacts caused by chemical insecticides in our environment. Thus, this current research provides evidential framework for* A. pinnata* as potential larvicidal agents. Furthermore, its application may alter the morphology and behavioural process of* Ae. aegypti* larvae. Therefore, it can be concluded that* A. pinnata* has desired compounds in larvicidal bioassays and future initiatives are necessary to investigate its bioactive compounds.

## Figures and Tables

**Table 1:** Larvicidal activity of *Aedes aegypti* using *Azolla pinnata* powdered form and fresh plant.

**Time**	**Treatment**	**LC**_**50**_ ** (ppm)** **with 95% confidence interval**	**LC**_**90**_ ** (ppm)** **with 95% confidence interval**	**Regression equation**
24 hours	Powdered *Azolla pinnata*	1262.794 (1030.588-1928.775)	1853.238 (1030.588-1928.775)	Y= 7.692X – 23.857
	Fresh *Azolla pinnata*	192517.205 (111397.462-478710.318)	2521535.166 (855514.813-19888028.270)	Y= 1.147X – 6.062

LC_50_, lethal concentration required to kill 50% of the population exposed; LC_90_, lethal concentration required to kill 95% of the population exposed and ppm, parts per million.

**Table 2:** Analysis of variance on* Aedes aegypti* larval mortality between powdered and fresh *Azolla pinnata* exposure for 24 hours.

Source of variation	df	MS	F-value	P-value
Concentration	1	7622200694.444	30.439	**p≤0.001** **∗**
Mortality	1	532.900	20.002	**p≤0.001** **∗**

df, degree of freedom; MS, mean-squared value.

Significant values are given in bold.

## Data Availability

The data used to support the findings of this study are available from the corresponding author upon request.

## Supplementary Materials

Supplementary Materials Visualization video on behavioural response effects of* Ae. aegypti* late third-stage larvae after 24-hour bioassay test in powdered and fresh* Azolla pinnata*. The movement of larvae is inactive from both observations with the presence of whitish pigmented cuticles.
